# Acetyl peroxy radical-initiated oxidation of oxygenated monoterpenes: functional group effects on reaction pathways

**DOI:** 10.1039/d5ea00117j

**Published:** 2025-12-01

**Authors:** Ida Karppinen, Dominika Pasik, Nanna Myllys

**Affiliations:** a Department of Chemistry, University of Helsinki Helsinki 00014 Finland nanna.myllys@helsinki.fi; b Institute for Atmospheric and Earth System Research, University of Helsinki Helsinki 00014 Finland

## Abstract

Biogenic volatile organic compounds are emitted into the atmosphere where they can oxidize forming compounds with lower volatilities. These low-volatility compounds can participate in the formation of secondary organic aerosols (SOAs) that affect human health and the climate in various ways. We studied the oxidation pathways initiated by the acetyl peroxy radical (APR) of less frequently studied oxygenated monoterpenes (monoterpenoids) using computational methods. The studied reactions included APR-addition, ring-opening reactions and C–C bond scissions. Ring-rearrangement after APR-addition was shown to be significant (43% yield) for one bicyclic monoterpenoid, sabinol. All other studied monoterpenoids will mostly react only with O_2_ forming a peroxy radical which will go on to form an alkoxy radical. Alkoxy radical β-scissions lead to different kinds of products depending on the reacting monoterpenoid. Verbenol and α-terpineol will mostly form closed-shell species through alkoxy radical C–C bond scissions leading to low SOA yields. The fate of carveol depends strongly on the stereoisomer. The *S*-isomer will form a closed-shell species with a 50% yield, whereas the *R*-isomer will form an alkyl radical with a 100% yield capable of further oxidation. Therefore, a significant SOA yield could be expected for carveol depending on the stereoisomer. For sabinol, umbellulone and carvone, high SOA yields are expected as the majority of them will form an alkyl radical that reacts with O_2_ forming a new peroxy radical. In the case of umbellulone and carvone, the formed peroxy radical is an acyl peroxy radical which can undergo rapid unimolecular reactions initiating fast autoxidation. Compared to the monoterpene counterparts of the studied monoterpenoids, significant differences were observed in reaction pathways and SOA yields. While APR-initiated oxidation serves as a minor pathway in the atmosphere, the studied reactions could have an impact on the production of low-volatility compounds.

## Abstract 2

Monoterpenoid oxidation by the acyl peroxy radical can lead to a termination reaction or further autoxidation and high secondary organic aerosol yield.

## Graphical abstract

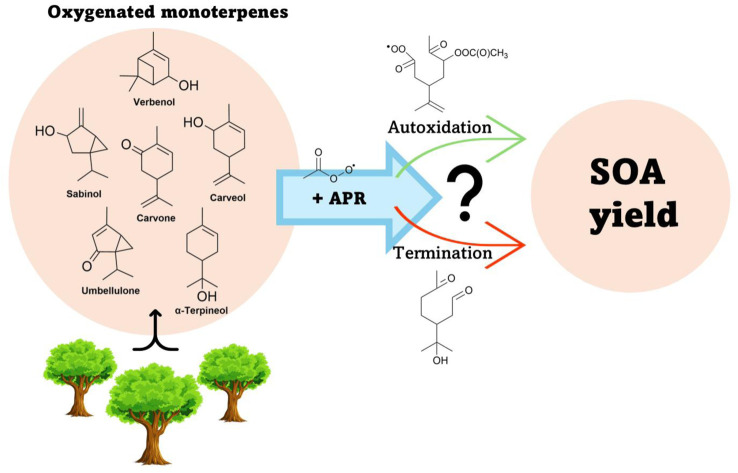

**Environmental significance**

We demonstrate that acyl peroxy radical-initiated oxidation of monoterpenoids is a relatively fast reaction in the atmosphere. Our results showcase that depending on the monoterpenoid structure, its oxidation can lead to a rapid termination reaction or further autoxidation and highly oxygenated organic molecule formation. This means the biogenic secondary organic aerosol (BSOA) yield is highly dependent on the exact monoterpenoid structure. Emissions of sabinol, umbellulone, carvone and carveol are likely to lead to high BSOA yield which is known to have negative radiative forcing. This is an important finding to improve existing models and also for policy makers to regulate biogenic emissions and further BSOA formation.

## 1 Introduction

Volatile organic compounds (VOCs) are emitted into the atmosphere, the majority of which are from biogenic sources.^1^ Most of the emitted VOCs undergo degradative oxidation initiated by hydroxyl radicals (as well as NO_3_ and O_3_) leading to the formation of CO_2_ and water.^2^ However, a small fraction will oxidize forming larger compounds with low volatilities. These low-volatility organic compounds (LVOCs) can condense onto already existing particles or create new ones through new particle formation (NPF) and participate in the formation of secondary organic aerosols (SOAs).^3,4^ Depending on the location, 20–90% of the aerosol mass is organic matter of which the largest fraction is usually secondary in nature.^5–7^ Aerosols are known to have impacts on human health, air quality as well as climate, including a cooling role in radiative forcing through cloud formation.^8–11^ The magnitude of the effect of aerosols on radiative forcing is however very uncertain.^11^ Therefore, the understanding of the small fraction of VOCs that oxidizes forming larger molecules is important.

A significant portion (up to 50%) of global SOA formation could be attributed to biogenic VOC (BVOC) oxidation.^12^ Approximately 15% of the global BVOC emissions comprise monoterpenes of which a large portion constitutes α-pinene, β-pinene and limonene.^1,13^ However, regional emissions vary and some globally negligible monoterpenes, *e.g.* oxygenated monoterpenes, could have significant effects on local SOA production.^14,15^ Moreover, climate change and the increase in temperature can lead to an enhancement of BVOC emissions from plants and even changes in BVOC profiles.^16,17^ This increases the need to understand the oxidation processes of less abundant but possibly more reactive monoterpenes. The SOA yields of oxygenated monoterpenes (from here on called monoterpenoids) have been the focus of a few studies.^15,18–23^ The presence of oxygen containing functional groups in monoterpenoids can lead to significant differences in SOA yields compared to monoterpenes which is why there is need for more studies surrounding this topic.

Acyl peroxy radicals have been shown to react fast enough with unsaturated hydrocarbons, *e.g.* monoterpenes, and these reactions can occur under atmospheric conditions.^24,25^ Although this is a minor pathway, these reactions could lead to the formation of low vapor pressure compounds. Reactions between acyl peroxy radicals and monoterpenes lead to the formation of an alkyl radical that rapidly reacts with O_2_ forming a peroxy radical.^26^ This peroxy radical can react further unimolecularly forming more oxidizied products or bimolecularly with radicals such as RO_2_ or HO_2_ forming an alkoxy radical. Alkoxy radical C–C bond scissions (β-scissions) can initiate autoxidation or the formation of closed-shell products that terminate the oxidation channel. Autoxidation is a key mechanism in which peroxy radicals react rapidly through subsequent intramolecular H-shift reactions to form highly oxygenated organic molecules (HOMs) with very low volatilities.^27–29^ Numerous studies have shown that HOMs are important for SOA growth.^30–32^ It is highly dependent on the alkoxy radical structure, in which the β-scission channel is the most favorable.^33–35^ Thus, the SOA yields from these different channels differ greatly depending on the parent alkoxy radical, *i.e.* the initial reacting monoterpene.

**Fig. 1:**
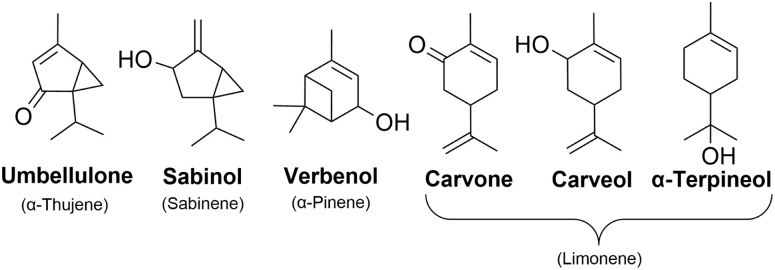
Structures of monoterpenoids investigated in this study. In brackets are the monoterpene counterparts of each monoterpenoid that were studied by Pasik *et al.*^35^

**Fig. 2:**
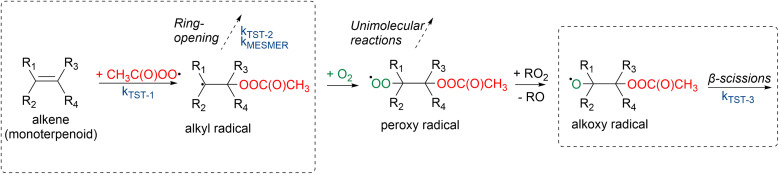
Monoterpenoid + APR reactions. Reaction rate coefficients were calculated for the circled reactions.

Draper *et al.*^34^ studied the oxidation pathways of monoterpenes initiated by the NO_3_ radical, and later on Pasik *et al.*^35^ studied similar oxidation pathways initiated by the acetyl peroxy radical (from here on abbreviated APR) of 7 different monoterpenes. Their studies showed significant differences in reaction pathways and SOA yields depending on the reacting monoterpene. This study builds upon the work by Pasik *et al.*^35^ to monoterpenoids, where oxygen containing functional groups are present. Using computational methods, we investigated the APR-initiated oxidation of six monoterpenoids: umbellulone, carvone, carveol, sabinol, verbenol and α-terpineol. The structures of the studied monoterpenoids are shown in Fig. 1 alongside their monoterpene counterparts that were studied by Pasik *et al.*^35^ Addition of APR is often followed by O_2_ addition, however, in the case of bicyclic monoterpenoids, alkyl-radical ring-opening reactions can be competitive.^34,35^ Therefore, the studied reactions included APR-addition, ring rearrangement and β-scission reactions of the alkoxy radical that forms after O_2_ addition as shown in Fig. 2. The goal of this study was to determine how the APR-initiated oxidation pathways and possible SOA yields are affected by functional groups, and how these compare to the monoterpene counterparts.

## 2 Methods

The methodology described here is adopted from the work by Pasik *et al.*,^36^ where a cost-effective and accurate methodology was developed to study reactions between unsaturated hydrocarbons and oxygenated organic radicals. Transition state (TS) structures corresponding to addition reactions between APR and monoterpenoids as well as subsequent ring-opening and β-scission reactions were located using the relaxed potential energy surface (PES) scan using density functional theory (DFT). All reactant, TS and product structures were optimized using DFT. TS structures were identified as correct with the presence of one imaginary frequency and by performing intrinsic reaction coordinate (IRC) calculations to verify that the TS connects to the correct reactant and product wells. All DFT calculations were carried out at the ωB97X-D/6-31+G* level of theory.^37–40^ Conformational sampling of reactant, TS and product structures was conducted using Conformer-Rotamer Ensemble Sampling Tool (CREST) at the GFN2-*x*TB level.^41,42^ Conformational sampling of TS structures included constraining the bonds being formed/broken. Conformers were optimized with DFT using a 2.5 kcal mol^−1^ cutoff after CREST, as *x*TB level electronic energies have shown to correlate fairly well with DFT energies.^36,43^ TS conformers were first optimized keeping the bond being formed/broken frozen, followed by full TS optimization and frequency calculation. Duplicates were removed based on electronic energy and dipole moment. For the lowest energy reactant, TS and product conformers, single-point energies were calculated on top of the ωB97X-D/6-31+G*-optimized structures at the DLPNO-CCSD(T)/aug-cc-pVTZ level of theory for more accurate estimation of reaction energy barriers.^44–47^ DFT calculations were performed using Gaussian 16 revision C.02, and single-point energies were calculated using ORCA version 5.0.3.^48,49^

Reaction rate coefficients were calculated using Multi-Conformer Transition State Theory (MC-TST).^50^ Rate coefficients for bimolecular addition reactions between APR and monoterpenoids were calculated using eqn (1):^51,52^1
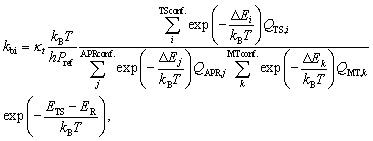
where *κ*_*t*_ is the quantum-mechanical tunneling coefficient, which is assumed to be 1 for reactions involving atoms other than hydrogen, *k*_B_ is the Boltzmann's constant, *T* is the temperature (=298.15 K), *h* is the Planck's constant and *P*_ref_ is the reference pressure (=2.45 × 10^19^ mol cm^−3^). Δ*E*_*i*_, Δ*E*_*j*_ and Δ*E*_*k*_ are the zero-point corrected energies of the TS, APR and monoterpenoid conformers, respectively, relative to the lowest energy conformer and *Q*_TS,*i*_, *Q*_APR,*j*_ and *Q*_MT,*k*_ are the partition functions of TS, APR and monoterpenoid conformers. *E*_TS_ and *E*_R_ are the zero-point corrected energies of the lowest energy TS and reactant conformers, respectively, including the DLPNO-CCSD(T)/aug-cc-pVTZ correction.

Reaction rate coefficients for unimolecular ring-opening and β-scission reactions were calculated using eqn (2):^53^2
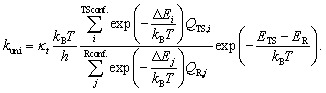


The dynamics of monoterpenoid-derived alkyl radical ring-opening reactions were studied using the methodology described in the work by Draper *et al.*,^34^ and later also used to study APR + monoterpene-derived alkyl radical ring-opening reactions in the work by Pasik *et al.*^35^ For two bicyclic monoterpenoids (sabinol and verbenol), the ring-opening reactions were simulated using the Master Equation Solver for Multi Energy-Well Reactions (MESMER) software.^54^ Vibrational frequencies and rotational constants were calculated using DFT level ωB97X-D/6-31+G*. The zero-point corrected energies were calculated at the DLPNO-CCSD(T)/aug-cc-pVTZ//ωB97X-D/6-31+G* level. APR-addition reactions were modeled assuming a barrierless association using the Inverse Laplace Transform (ILT) method. MC-TST calculated reaction rate coefficients for APR-addition reactions were used as a pre-exponential factor in the ILT calculations. O_2_-addition reactions were treated as bimolecular sinks using a bimolecular rate coefficient of 6 × 10^−12^ cm^3^ molecule^−1^ s^−1^ and O_2_ concentration of 5.16 × 10^18^ mol cm^−3^, values previously used in other studies.^33,35,55^ Ring-opening reactions were modeled using standard Rice–Ramsperger–Kassel–Marcus (RRKM) theory with Eckart tunneling correction. RRKM theory takes into account the excess energy released in the APR-addition reaction which affects the reaction rate coefficient of the ring-opening. An exponential-down parameter of *E*_down_ = 225 cm^−1^ was used to model collisional energy transfer. The bath gas used was N_2_ with Lennard–Jones parameters *ε*/*k*_B_ = 91.85 K and *σ* = 3.919 Å. The Lennard–Jones parameters for monoterpenoid-derived alkyl radicals and ring-opened products, which are available in the SI (Table S1), were obtained based on the group-additivity method applied to pure-compound critical properties by Joback and Reid,^56^ a procedure described in Gao *et al.*^57^ and Tee *et al.*^58^ A concentration of 10^15^ mol cm^−3^ was used for APR to guarantee the rapid formation of the modeled monoterpenoid-APR intermediate. A grain size of 30 cm^−1^ was used, and the simulated energy grains spanned 50*k*_B_*T* above the highest TS.

## 3 Results

### 3.1 Acetyl peroxy radical addition reactions

We started by investigating the kinetics of reactions between APR and monoterpenoids shown in Fig. 1. We considered all relevant APR-addition sides and calculated rates for addition reactions to double bonds. The rate is affected by stereochemistry, which was also considered. For umbellulone, carveol, sabinol and verbenol, we calculated APR-addition rates for different sides, where the attacking APR can either be on the same side of the secondary ring structure/OH-group, or on the opposite side. It was previously shown that it indeed affects the rate when considering different sides of APR attack relative to the secondary ring structure.^35^ The fastest calculated reaction rate coefficients for APR-addition reactions are listed in Table 1. Other calculated rate coefficients can be found in the SI (Table S2). Addition reactions are labeled according to the side of the attacking APR: (a) APR attack from the secondary ring side, (b) attack from the methyl group side, (c) attack from the OH-group side, and (d) attack from the side opposite to the OH-group. In cases where both the secondary ring and the OH-group are present, combinations such as (a,d) indicate that attack occurs from the same side of the secondary ring but from the opposite side of the OH-group.

**Table 1:** Calculated energy barrier heights (Δ*E*_1_ in kcal mol^−1^), bimolecular MC-TST reaction rate coefficients (*k*_TST-1_ in cm^−3^ s^−1^) and excess energy (Δ*E*_ex_ in kcal mol^−1^) for the fastest APR + monoterpenoid addition reactions at 298 K. Δ*E*_2_, *k*_TST-2_ and *k*_MESMER_ are the calculated energy barrier heights (in kcal mol^−1^), MC-TST and RRKM-ME rate coefficients (in s^−1^) for the ring-opening reactions of monoterpenoid-derived alkyl radicals, respectively. %_TST-2_ and %_MESMER_ are MC-TST and RRKM-ME yields for ring-opening reactions. MC-TST yields were calculated assuming a rate coefficient of 6 × 10^−12^ cm^3^ molecule^−1^ s^−1^ with a O_2_ concentration of 5.16 × 10^18^ mol cm^−3^ and RRKM-ME yields were taken from time profiles. Addition reactions are labeled according to the side of the attacking APR: (a) APR attack from the secondary ring side, (b) attack from the methyl group side, (c) attack from the OH-group side, and (d) attack from the side opposite to the OH-group

Monoterpenoid	Δ*E*_1_	*k*_TST-1_	Δ*E*_ex_	Δ*E*_2_	*k*_TST-2_	*k*_MESMER_	%_TST-2_	%_MESMER_
*α*-Terpineol	0.9	7.9 × 10^−17^	16.8	✗	✗	✗	✗	✗
Umbellulone (b)	1.7	9.5 × 10^−18^	16.5	✗	✗	✗	✗	✗
Carveol (c)	−0.1	1.3 × 10^−15^	15.8	✗	✗	✗	✗	✗
Carvone	1.5	8.0 × 10^−17^	17.5	✗	✗	✗	✗	✗
Sabinol (b,c)	−0.8	4.9 × 10^−16^	17.6	10.0	7.1 × 10^6^	6.4 × 10^5^	19	43
Verbenol (a,c)	1.3	1.1 × 10^−16^	14.6	14.0	2.3 × 10^2^	2.4 × 10^2^	∼ 0	∼ 0
Verbenol (a,d)	1.9	1.2 × 10^−16^	14.7	13.2	1.7 × 10^3^	5.3 × 10^3^	∼ 0	1

Considering the stereochemistry has a substantial impact on the calculated barriers. The rate is affected by both the attack side relative to the secondary ring side and OH-group side. For carveol, the APR attack happening from the OH-group side exhibits 1.8 kcal mol^−1^ lower barrier than the APR attack happening from the opposite side of the OH-group. When the OH-group lies on the same side of the attacking APR, hydrogen bonding can occur, lowering the reaction barrier. The same is observed for sabinol, where the attack happening from the same side of the OH-group and methyl group has the lowest barrier.

For verbenol, attacks from both the OH-group side (c) and the opposite side (d) yield similar reaction rate coefficients, even though attack occurring from the OH-group side has a 0.6 kcal mol^−1^ lower barrier. This results from a different amount of unique TS conformers: the OH-group side has 3 unique TS conformers, whereas the opposite side has 18. The larger number of conformers increases the overall partition function sum in eqn (1), compensating for the barrier difference. Consequently, the rate coefficients for different sides of APR attack are similar even though the reaction barriers differ. The same was also observed for verbenol in the APR-addition reaction that produces a secondary alkyl radical. Attack happening from the OH-group side exhibited 1.9 kcal mol^−1^ lower barrier than attack happening from the opposite side, however, the rates for these reactions were of the same order of magnitude (see SI Table S2). This was again due to different amounts of unique TS conformers (2 for reaction c and 15 for reaction d).

The fastest addition reaction results in the formation of a tertiary radical for all monoterpenoids studied, except for umbellulone. For umbellulone, the fastest addition reaction involves the formation of a secondary alkyl radical that is stabilized by the adjacent carbonyl group. The formation of a tertiary radical would hinder the conjugation in umbellulone which makes this reaction pathway less favorable. For the pathway that involves the formation of a secondary radical, addition b is favored. However, for the pathway involving the formation of a tertiary radical, addition a is favored (see SI Table S2). For this secondary radical forming pathway, addition to the cyclopropyl side (a) has a barrier as high as 7.1 kcal mol^−1^. We conducted additional conformer sampling for the TS using the new Global Optimizer GOAT in Orca 6.0.^59^ However, conformer search using GOAT resulted in the same lowest energy TS conformer as configurational search using CREST. Further details and discussion are provided in the SI.

Fastest addition reactions are observed for carveol, sabinol and verbenol which have an OH-group in the structure. When compared to results obtained by Pasik *et al.*^35^ on APR-initiated monoterpene oxidation, sabinol and carveol exhibit lower barriers than their monoterpene counterparts sabinene and limonene, respectively. The reaction between APR and α-terpineol has a slightly lower barrier than the APR + limonene reaction. However, in the case of limonene, the reaction rate coefficient is approximately an order of magnitude higher. Verbenol and its monoterpene counterpart α-pinene have similar barriers and reaction rate coefficients. Contrastingly, carvone and umbellulone exhibit higher barriers and slower reactions than their monoterpene counterparts limonene and α-thujene, respectively. Evidently, the OH-group near the APR attack site lowers the reaction barrier, whereas a carbonyl group near the APR attack site increases the barrier. Altogether, reaction barriers were low and all reaction rate coefficients in Table 1 would be considered high enough for these reactions to be feasible under atmospheric conditions.

There is very limited data available for the OH-initiated oxidation of the investigated monoterpenoids. The gas-phase reaction between α-terpineol and OH has been studied experimentally and a rate of 1.9 ± 0.5 × 10^−10^ cm^3^ molecule^−1^ s^−1^ was reported.^60^ With an average atmospheric OH concentration of 10^6^ mol cm^−3^,^61^ this rate would correspond to a pseudo-first-order reaction rate of 1.9 × 10^−4^ s^−1^. The total reaction rate coefficient for α-terpineol + APR reaction is a sum of the two rates calculated here (see Tables 1 and S2) and gives a rate of 9.1 × 10^−17^ cm^3^ molecule^−1^ s^−1^. Using a concentration of 10^8^ mol cm^−3^ for APR,^62^ a pseudo-first-order reaction rate of 9.1 × 10^−9^ s^−1^ is derived for the α-terpineol + APR reaction. Thus, the APR-initiated oxidation would correspond to a negligible percentage of the OH-initiated oxidation of α-terpineol. For the other monoterpenoids, such a comparison is not possible due to the lack of experimental data on OH-initiated oxidation of said structures.

There is uncertainty in our estimation of absolute rate coefficients that could lead to underestimation of rates of the APR-initiated oxidation of monoterpenoids. In our previous study,^63^ we investigated the reaction between APR and isoprene and comparison to experimental data presented by Nozière and Fache^24^ and SAR predictions by Stark^64^ showed an underestimation of one to two orders of magnitude in our calculated rate. Accordingly, we are assuming a similar underestimation of the absolute rates of reactions between APR and monoterpenoids reported here. Theoretical reaction rates are usually associated with an uncertainty of approximately a factor of five arising from the sensitivity analysis where barrier heights are systematically varied by ± 1 kcal mol^−1^. Even with this uncertainty, the computed rates for isoprene + APR reactions remain lower than the experimental values. Sources of uncertainty could arise from the larger uncertainties introduced by approximations used in the DLPNO-CCSD(T) approach compared to canonical CCSD(T). However, for the large systems studied here, canonical CCSD(T) is too expensive. The uncertainties discussed here could lead to an underestimation of the contribution of APR-initiated oxidation in monoterpenoid oxidation. Recent experiments have verified that APR + double bond chemistry indeed occurs under laboratory conditions.^25,65^ However, more experimental data would be needed to confirm reaction kinetics and branching ratios.

### 3.2 Monoterpenoid-derived alkyl radical ring-opening reactions

After APR-addition, O_2_-addition to the alkyl radical center is expected due to its high concentration in the atmosphere. However, the excess energy released in the APR-addition reaction can help overcome the barrier for subsequent ring-opening reactions. Thus, we further studied the ring-opening reactions following APR-addition for sabinol and verbenol using two different methods, MC-TST and RRKM simulations. No ring-opening reaction is expected for umbellulone as the radical center is too far from the secondary ring structure. Verbenol exhibits slow ring-opening reactions and the ring-opening is a minor (1%) pathway for the verbenol + APR d-adduct and negligible for the c-adduct. This was expected as the secondary ring was a four-membered ring, and ring-opening reactions have shown to become more competitive as the ring size decreases.^34,35^ Compared to α-pinene, the monoterpene counterpart of verbenol studied by Pasik *et al.*,^35^ the yield of the alkyl radical ring-opening reaction is enhanced only slightly (0% for α-pinene and 0–1% for verbenol).

**Fig. 3:**
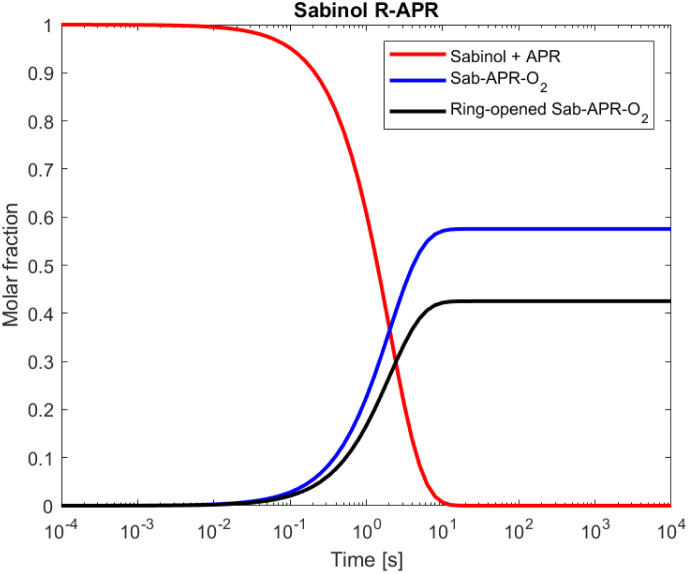
Time profile for the bicyclic sabinol-derived alkyl radical reacting *via* ring-opening *versus* O_2_ addition simulated using RRKM-ME. Red line corresponds to the reactant (sabinol + CH_3_C(O)OȮ) for the ring-opening reaction, black line represents the product of O_2_ addition to the ring-opened alkyl radical, and the blue line corresponds to the non-ring-opened product (sabinol + CH_3_C(O)OȮ) that undergoes O_2_ addition.

For sabinol, which has a three-membered secondary ring, the ring-opening reaction becomes much more significant due to the small and strained ring-structure. RRKM simulations show a 43% yield for the ring-opening reaction, whereas MC-TST calculations predict a significantly lower yield of 19%. Interestingly, the MESMER-derived rate coefficient is lower than the MC-TST rate coefficient. MESMER-derived rates only account for one conformer which could explain the lower rate coefficient. Despite the lower rate coefficient, RRKM simulations still result in a larger yield for ring-opening reaction for sabinol. The simulated time profile of species distribution for sabinol-derived alkyl radical ring-opening *versus* O_2_ addition is shown in Fig. 3. The yield of the sabinol-derived alkyl radical ring-opening reaction is also larger compared to the yield of its monoterpene counterpart sabinene (31%) studied by Pasik *et al.*^35^

### 3.3 Monoterpenoid-derived alkoxy radical β-scission reactions

**Fig. 4:**
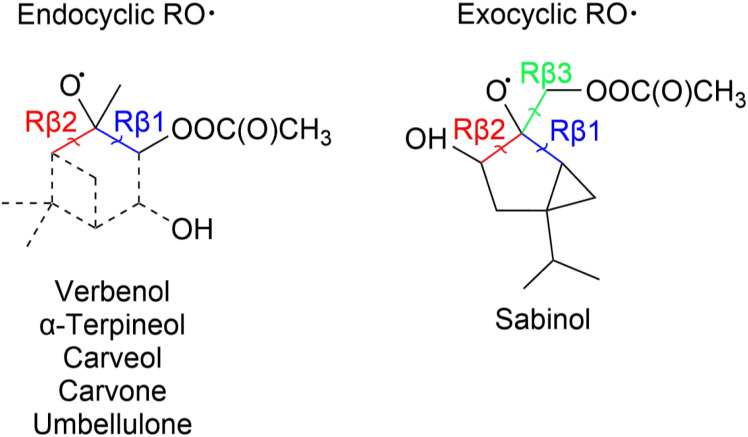
Reaction labelling used for acetyl-alkoxyl β-scission reactions.

After the addition of APR to the monoterpenoid, the formed product can rapidly react with O_2_ to form a peroxy radical. The peroxy radical can again react to form an alkoxy radical as shown in Fig. 2. This alkoxy radical can undergo β-scission reactions that were studied for all monoterpenoids. “Right” (Rβ1) and “left” (Rβ2) β-scissions were studied for the endocyclic alkoxy radicals and “right”, “left” and “top” (Rβ3) β-scissions were studied for the exocyclic alkoxy radical as shown in Fig. 4. Stereochemistry was also taken into account according to Fig. S4. Calculated energy barriers, MC-TST rate coefficients and product yields for the different β-scission channels are given in Table 2.

**Table 2:** Calculated energy barrier heights (Δ*E* in kcal mol^−1^), unimolecular MC-TST reaction rate coefficients (*k*_TST-3_ in s^−1^) and product yields for acetyl-alkoxyl β-scission reactions

Monoterpenoid	Δ*E*			*k*_TST-3_			Yield (%)		
	Rβ1	Rβ2	Rβ3	Rβ1	Rβ2	Rβ3	Rβ1	Rβ2	Rβ3
Carveol (*R*)	9.5	2.3	✗	9.5 × 10^5^	1.0 × 10^11^	✗	0	100	✗
Carveol (*S*)	4.9	4.3	✗	2.0 × 10^9^	2.0 × 10^9^	✗	50	50	✗
Carvone (*R*,*R*)	6.3	1.3	✗	1.0 × 10^8^	4.4 × 10^11^	✗	0	100	✗
Carvone (*R*,*S*)	7.6	0.8	✗	1.3 × 10^7^	2.0 × 10^12^	✗	0	100	✗
Carvone (*S*,*R*)	6.9	0.1	✗	1.2 × 10^7^	3.5 × 10^12^	✗	0	100	✗
Carvone (*S*,*S*)	3.7	−0.6	✗	2.9 × 10^9^	7.0 × 10^12^	✗	0	100	✗
α-Terpineol (*R*,*R*)	10.2	11.4	✗	4.8 × 10^5^	3.3 × 10^4^	✗	93	7	✗
α-Terpineol (*R*,*S*)	6.3	11.9	✗	5.1 × 10^7^	1.7 × 10^4^	✗	100	0	✗
α-Terpineol (*S*,*R*)	6.4	10.5	✗	1.7 × 10^7^	3.5 × 10^4^	✗	100	0	✗
α-Terpineol (*S*,*S*)	8.1	12.6	✗	2.2 × 10^6^	3.9 × 10^3^	✗	100	0	✗
Umbellulone (*R*)	7.9	1.5	✗	3.0 × 10^7^	1.1 × 10^12^	✗	0	100	✗
Umbellulone (*S*)	7.8	1.7	✗	1.5 × 10^7^	1.1 × 10^12^	✗	0	100	✗
Sabinol (*R*,*R*)	0.7	17.5	10.3	4.7 × 10^11^	2.5 × 10^0^	4.9 × 10^4^	100	0	0
Sabinol (*R*,*S*)	2.2	15.0	9.8	4.6 × 10^10^	4.8 × 10^2^	4.7 × 10^5^	100	0	0
Sabinol (*S*,*R*)	0.8	16.3	6.9	7.8 × 10^11^	2.1 × 10^2^	2.0 × 10^8^	100	0	0
Sabinol (*S*,*S*)	0.03	12.8	7.3	2.9 × 10^12^	1.4 × 10^4^	3.5 × 10^7^	100	0	0
Verbenol (*R*,*R*)	8.5	11.5	✗	7.2 × 10^6^	4.0 × 10^4^	✗	99	1	✗
Verbenol (*R*,*S*)	8.9	12.2	✗	1.8 × 10^6^	1.3 × 10^4^	✗	99	1	✗
Verbenol (*S*,*R*)	6.7	12.6	✗	4.1 × 10^7^	6.7 × 10^3^	✗	100	0	✗
Verbenol (*S*,*S*)	6.8	11.2	✗	9.4 × 10^7^	8.0 × 10^4^	✗	100	0	✗

β-Scission channels resulting in the formation of a radical center on a three- or four-membered ring structure have the highest energy barriers which has also been observed for monoterpene derived nitrooxy- and acetyl-alkoxyl β-scissions.^34,35^ High barriers are also observed for the α-terpineol Rβ2 channel which forms a primary alkyl radical. Channels leading to the formation of an acyl radical or α-OH alkyl radical center exhibit the lowest barriers (as low as −0.6 kcal mol^−1^). The channel where an acyl radical forms is especially interesting as rapid reaction with O_2_ leads to the formation of a new acyl peroxy radical that can undergo fast unimolecular reactions initiating possible further autoxidation. Each monoterpenoid has its own unique reactivity and although most stereoisomers of the parent monoterpenoid exhibit similar reactivity, some differences are also observed between different isomers. As a result, each monoterpenoid has a different potential to contribute to the formation of SOA. A more detailed description of each monoterpenoid's reactivity and a proposed fate is given in the following sections.

#### 3.3.1 Carveol

**Fig. 5:**
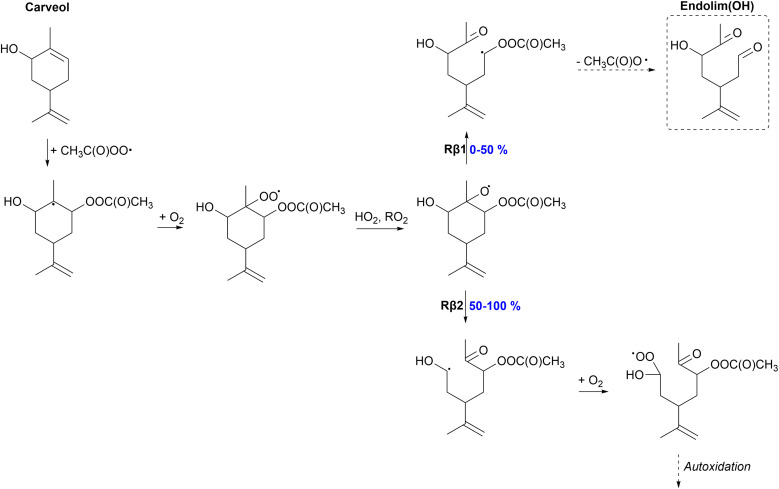
Proposed fate of carveol + CH_3_C(O)OȮ. Dashed box indicates termination of the oxidation pathway through the formation of a closed-shell product.

The proposed fate of the APR-initiated oxidation of carveol is shown in Fig. 5. The addition reaction between APR and carveol results in the formation of a tertiary alkyl radical which rapidly reacts with O_2_ forming a peroxy radical. This peroxy radical most likely undergoes a bimolecular reaction and an alkoxy radical is formed. However, the peroxy radical can also react unimolecularly through H-shift reactions. Different H-shift reactions were studied for a similar limonene-derived peroxy radical, the monoterpene counterpart of carveol, by Pasik *et al.*^35^ Their calculations showed that the H-shift reactions for the limonene-derived peroxy radical are slow, the fastest H-shift being an allylic 1,6 H-shift with a rate of 1 × 10^−3^ s^−1^. In another study by Møller *et al.*,^66^ the H-shifts of a limonene-derived hydroxy peroxy radical, similar to the carveol-derived peroxy radical, were studied computationally. These H-shifts were also slow, the fastest being the hydrogen abstraction from the OH-group with a rate of 4 × 10^−4^ s^−1^ for the *S*-isomer (OH- and peroxy group on the same side) and even slower for the *R*-isomer. Based on these studies, we are assuming that the carveol-derived peroxy radical does not react unimolecularly and reacts bimolecularly forming the alkoxy radical.

Unlike other monoterpenoids, carveol shows significant differences in the preferred β-scission pathways depending on the stereoisomer. Of the *R*-isomer, 100% will react through the left C–C scission (Rβ2) forming the α-OH alkyl radical. The yields of Rβ1 and Rβ2 channels for the *S*-isomer are both 50%. Right C–C scission (Rβ1) leads to the formation of a α-QOOR alkyl radical which is unstable and undergoes decomposition as shown in other studies.^28,67,68^ Therefore, this pathway will lead to the formation of a closed-shell species, endolim(OH), and termination of the oxidation chain. The formed α-OH alkyl radical in the Rβ2 channel could add O_2_ and react unimolecularly leading to more oxidized products, and eventually HOMs, exhibiting higher SOA yields. In the APR-initiated oxidation of limonene, the formed alkoxy radical reacts 100% through the Rβ1 channel leading to the termination of the oxidation chain and low SOA yields.^35^ The SOA yield is possibly enhanced for carveol compared to limonene but it strongly depends on the stereoisomer.

#### 3.3.2 Carvone

**Fig. 6:**
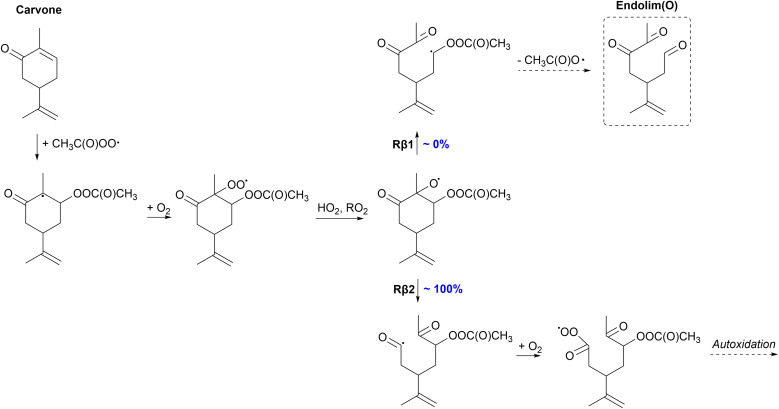
Proposed fate of carvone + CH_3_C(O)OȮ. Dashed box indicates termination of the oxidation pathway through the formation of a closed-shell product.

The mechanism for the oxidation of carvone is presented in Fig. 6. Like carveol, carvone first forms a tertiary alkyl radical in the APR addition step followed by O_2_ addition and the formation of a peroxy radical. We are also assuming that this formed peroxy radical will react bimolecularly forming an alkoxy radical rather than reacting unimolecularly as in the case of carveol. The formed alkoxy radical reacts through C–C bond scissions and clearly shows the preference of the Rβ2 channel (100%) for all stereoisomers. In this reaction channel, a highly stabilized acyl radical forms which makes it the more favoured channel. This channel exhibits extremely low barriers, as low as −0.6 kcal mol^−1^ for the *S*,*S*-isomer, and high reaction rates nearing 10^13^ s^−1^ which is very high (close to a theoretical maximum value) for a unimolecular C–C bond scission. The formed acyl radical presumably reacts with O_2_ forming an acyl peroxy radical. This acyl peroxy radical can react very rapidly unimolecularly initiating autoxidation which in turn can lead to the formation of HOMs and high SOA yields. The Rβ1 channel would lead to a closed-shell product, endolim(O), which terminates the oxidation chain leading to low SOA yields. However, the yield for this channel is 0% for all stereoisomers. Compared to limonene,^35^ carvone could have significantly higher SOA yields through the formation of an acyl peroxy radical that could initiate autoxidation. Presumably, the SOA yield for carvone would also be higher than that for carveol.

#### 3.3.3 α-Terpineol

**Fig. 7:**
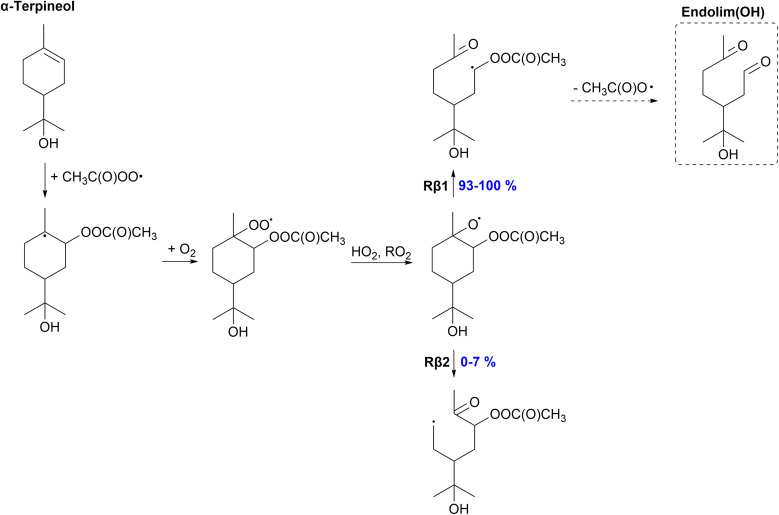
Proposed fate of α-terpineol + CH_3_C(O)OȮ. Dashed box indicates termination of the oxidation pathway through the formation of a closed-shell product.

The proposed fate of α-terpineol in the APR-initiated oxidation is shown in Fig. 7. A peroxy radical forms in the steps following the APR addition since there is no secondary ring structure present in α-terpineol. We do not expect fast unimolecular reactions for the α-terpineol-derived peroxy radical as monoterpene-derived hydroxy peroxy radicals with no double bonds have shown to react slowly through H-shift reactions.^66^ Therefore, an alkoxy radical will form from the peroxy radical which reacts through C–C bond scissions. The majority (93–100%) of C–C bond scissions will occur through the Rβ1 channel which leads to a closed-shell product and termination of the oxidation chain. The *R*,*R*-isomer shows a yield of 93% for the Rβ1 channel, whereas other isomers show a 100% yield. The Rβ2 channel leading to a primary alkyl radical centered product is minor (0–7%). O_2_ can add to the radical center followed by further oxidation. The formation of an alkyl radical is slightly enhanced for α-terpineol (0–7%) compared to limonene (0%).^35^ However, low SOA yields would be expected for α-terpineol, as the major pathway, Rβ2, leads to the termination of the oxidation chain.

#### 3.3.4 Umbellulone

**Fig. 8:**
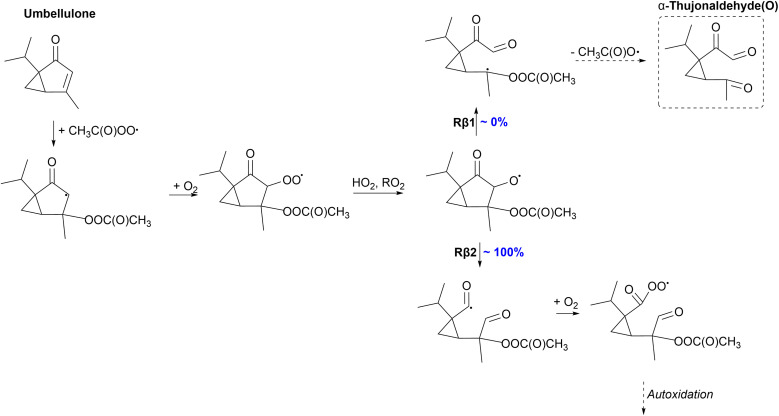
Proposed fate of umbellulone + CH_3_C(O)OȮ. Dashed box indicates termination of the oxidation pathway through the formation of a closed-shell product.

The oxidation mechanism of umbellulone initiated by APR is shown in Fig. 8. Umbellulone favors APR addition, where a secondary alkyl radical forms compared to all other monoterpenoids that favor the formation of a tertiary alkyl radical. The formed secondary alkyl radical is stabilized by the adjacent carbonyl group, which makes it more favorable. There is a secondary ring structure present in umbellulone; however, due to the preference to form a secondary alkyl radical, the radical center is too far from the secondary ring structure for an efficient ring-opening reaction. Therefore, O_2_ addition and the formation of a peroxy radical are expected. Unimolecular reactions of secondary ring containing monoterpenoid-derived RO_2_ radicals are expected to be slow due to the rigid structure and the formation of an alkoxy radical is favored. For umbellulone, 100% of the alkoxy radical reacts through the left C–C scission (Rβ2), where a highly stabilized acyl radical forms. This acyl radical can react with O_2_ forming a new acyl peroxy radical which can further react and oxidize to form HOMs. The right C–C scission (Rβ1) would lead to the formation of a closed-shell species, α-thujonaldehyde(O), and termination of the oxidation channel. Due to the formation of an acyl radical in the preferred Rβ2 channel, high SOA yields could be expected from the APR-initiated oxidation of umbellulone. α-Thujene, the monoterpene counterpart of umbellulone, reacts through the right C C scission leading to the termination of the oxidation channel with a yield of 100%.^35^ Accordingly, umbellulone would have a higher SOA yield compared to α-thujene.

#### 3.3.5 Sabinol

**Fig. 9:**
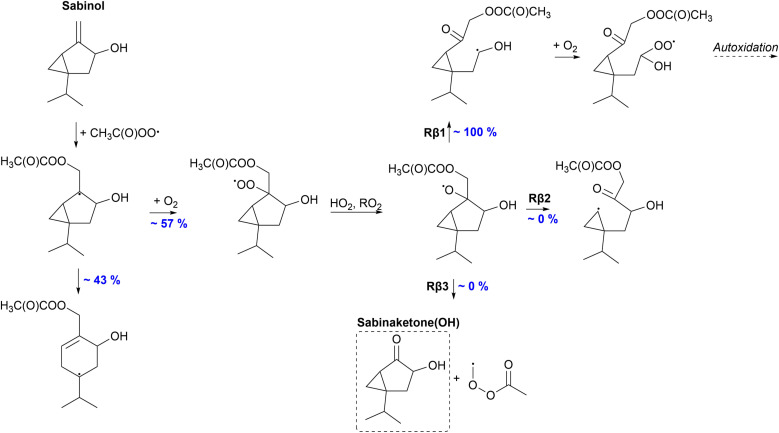
Proposed fate of sabinol + CH_3_C(O)OȮ. Dashed box indicates termination of the oxidation pathway through the formation of a closed-shell product.

The proposed fate of sabinol is presented in Fig. 9. As a bicyclic monoterpenoid, sabinol can react through the ring-opening reaction after APR addition. RRKM simulations predict a significant yield of 43% for the ring-opening reaction against O_2_ addition. An alkyl radical forms which can again react with O_2_ forming a peroxy radical. For a sabinene-derived ring-opened peroxy radical, the monoterpene counterpart of sabinol, a 1,5 H-shift has been shown to be relatively fast with a rate of 2 × 10^−2^ s^−1^.^35^ Accordingly, for the sabinol-derived ring-opened peroxy radical, H-shifts are expected to be as fast or even faster due to the presence of the OH-group that can enhance the rate.^69^ Fast H-shift reactions can initiate further oxidation and formation of low-volatility products that could participate in SOA formation.

57% of the formed alkyl radical will react through O_2_ addition eventually forming an alkoxy radical. In addition to left and right C–C scissions, top C–C scission (Rβ3) is possible for the sabinol-derived exocyclic alkoxy radical. However, according to our calculations, 100% of the alkoxy radical will react through the right C–C scission (Rβ1) forming an α-OH alkyl radical. Left-sided scission would form a radical center on a three-membered ring structure, whereas the top C–C bond scission terminates the oxidation chain through the formation of sabinaketone(OH). The α-OH alkyl radical formed in the Rβ1 channel can react with O_2_ and further oxidize, leading to low-volatility products. Both the ring-opening rearrangement and reaction with O_2_ of the initial alkyl radical can lead to the formation of HOMs predicting high SOA yields for the APR-initiated oxidation of sabinol. High SOA yield was also expected for the APR-initiated oxidation of sabinene by Pasik *et al.*,^35^ however, a significant percentage (31–63%) of the sabinene-derived alkoxy radical is shown to react through the top C–C scission leading to the termination of the oxidation chain. 100% of the sabinol-derived alkoxy radical will react through the Rβ1 channel that is most likely to initiate further oxidation and therefore higher SOA yields would be expected for sabinol than sabinene.

#### 3.3.6 Verbenol

**Fig. 10:**
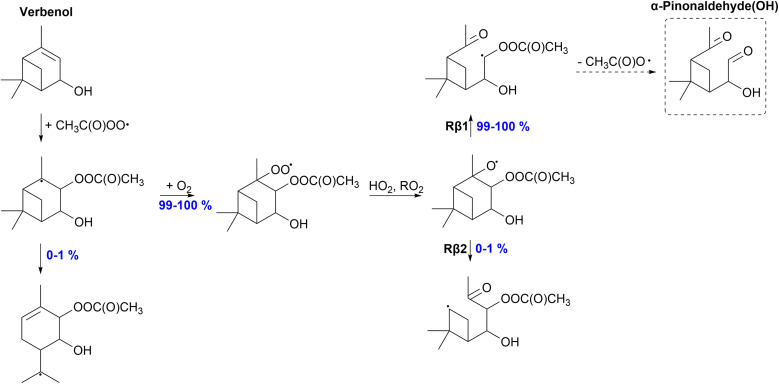
Proposed fate of verbenol + CH_3_C(O)OȮ. Dashed box indicates termination of the oxidation pathway through the formation of a closed-shell product.

The APR-initiated oxidation mechanism of verbenol is presented in Fig. 10. Addition of APR produces a bicyclic alkyl radical that can react through ring-opening rearrangement; however, this reaction is slow and only a minor (0–1%) yield is expected from this reaction. Thus, majority of the alkyl radical will form an alkoxy radical. Of this alkoxy radical, the majority (99–100%) will react through the right-sided C–C bond scission forming a α-QOOR alkyl radical that undergoes decomposition. A closed-shell species α-pinonaldehyde(OH) forms and terminates the oxidation chain. A 1% yield is shown for the *R*,*R*- and *R*,*S*-isomers for the Rβ2 channel. However, a low SOA yield is expected for verbenol according to our calculations. The same was observed for α-pinene, the monoterpene counterpart of verbenol.^35^

### 3.4 Atmospheric implications

The end products and expected SOA yields of all studied monoterpenoids are summarized in Table 3. The table includes the most likely oxidation products of each monoterpenoid and SOA yields predicted according to major oxidation pathways. Oxidation of carveol, carvone, umbellulone, and sabinol leads to the formation of peroxy radicals, which have potential to begin the autoxidation process that leads to the rapid formation of highly oxygenated organic molecules. These compounds tend to be very low volatile, thus capable of participating in secondary organic aerosol formation. In warm climates, biogenic VOC emissions increase. Depending on the type of emitting monoterpene or monoterpenoid, SOA yield can vary a lot. In the case of C_10_H_16_ monoterpenes, exocyclic monoterpenes lead to a higher SOA yield than endocyclic monoterpenes.^35^ For monoterpenoids, the functional group can lead to different branching of autoxidation/termination pathways. The more VOCs capable of autoxidizing are emitted, the more biogenic SOA is formed creating a negative climate feedback mechanism.^70^ Thus, emissions of α-terpineol or verbenol are likely to have minor impact to the climate forcing *via* SOA formation whereas, carveol, carvone, umbellulone, and sabinol could have larger impacts.

**Table 3:** Predicted end products and SOA yields of each studied monoterpenoid according to major oxidation pathways

Monoterpenoid	End product	SOA yield
Carveol	Endolim(OH) (0–50%)/HOM (50–100%)	Moderate/high
Carvone	HOM	High
α-Terpineol	Endolim(OH)	Low
Umbellulone	HOM	High
Sabinol	HOM (*via* two main pathways)	High
Verbenol	α-Pinonaldehyde(OH)	Low

Other acyl peroxy radicals (for which unimolecular reactions are slow) are known to react in a similar manner to the acetyl peroxy radical used as a representative APR in this study. Reactions of larger acyl peroxy radicals with unsaturated hydrocarbons are often faster compared to those of the acetyl peroxy radical,^25,63^ but the concentrations of these acyl peroxy radicals are even less well known. However, the larger acyl peroxy radicals, such as the benzoyl peroxy radical, when reacting with monoterpenoids can form compounds with more than 15 carbon atoms. These compounds (when highly oxygenated) can be classified as ultra-low-volatility organic compounds that can nucleate under atmospheric conditions.^71,72^ In contrast, self- and cross-reactions of RO_2_, where a ROOR dimer forms, are needed to form >C15 compounds from the OH-initiated oxidation. Thus, while APR-initiated oxidation serves as a minor pathway in the atmosphere, its impact on the production of low vapor pressure compounds should not be neglected. Moreover, the relevance of these reactions could be substantially enhanced in environments where reactions with OH are limited, *e.g.* at nighttime with low-NO_*x*_ and high-VOC conditions.

## 4 Conclusions

We investigated the oxidation pathways of six different oxygenated monoterpenes initiated by the acetyl peroxy radical. Our goal was to determine how oxygen containing functional groups affect the reaction pathways and SOA yields and compare our results with results obtained by Pasik *et al.*^35^ on APR-initiated oxidation of monoterpenes.

According to our calculations, APR-addition is enhanced by an OH-group near the APR attack site, whereas a carbonyl group will inhibit the reaction. The formation of a tertiary alkyl radical was favored for all but one monoterpenoid, umbellulone. For umbellulone, the APR-addition that forms a secondary alkyl radical is stabilized by the adjacent carbonyl group making it faster than the tertiary radical forming pathway. Ring rearrangement reactions were studied for verbenol- and sabinol-derived alkyl radicals. RRKM simulations showed that the yields of ring-opening reactions were negligible (0–1%) for verbenol, but significant (43%) for sabinol. Ring-opening yields were similar for verbenol and its monoterpene counterpart α-pinene^35^ (negligible for both), but notably enhanced for sabinol compared to the monoterpene counterpart sabinene (31%^35^). Sabinol-derived alkyl radical rearrangement could open up additional pathways for further oxidation leading to the formation of low-volatility compounds.

Each monoterpenoid has unique reactivity toward different C–C bond scission channels, leading to varying possibilities to initiate SOA formation. The formation of a closed-shell species in the APR-initiated oxidation is very likely for α-terpineol and verbenol suggesting low SOA yields. For carveol, the preferred β-scission depends strongly on the stereoisomer. The formation of a closed-shell species, endolim(OH), is negligible (0%) for the *R*-isomer but significant for the *S*-isomer (50%). 50–100% of carveol will react through the Rβ2 channel that forms an α-OH alkyl radical that could further oxidize. This would mean non-negligible SOA yield for carveol, but it is strongly dependent on the stereoisomer. High SOA yields would be expected for carvone and umbellulone through the formation of an acyl peroxy radical that could initiate fast autoxidation. Also, sabinol is expected to have high SOA yield in the APR-initiated oxidation. The SOA yield expectancy is similar for verbenol and α-terpineol compared to their monoterpene counterparts α-pinene and limonene, respectively, moderately higher for carveol compared to limonene and significantly higher for carvone, umbellulone and sabinol compared to their monoterpene counterparts limonene, α-thujene and sabinene, respectively.

Our study showcased significant differences in the oxidation mechanisms between monoterpenes and oxygenated monoterpenes. Oxygenated monoterpenes could have crucial effects on local SOA formation through enhanced reactivity toward oxidants. Consequently, understanding the oxidation mechanisms and SOA formation from these oxygenated monoterpenes is important.

## Author contributions

IK performed the calculations and wrote the manuscript; DP assisted with the calculations; DP and NM contributed to the analysis. The study was designed and supervised by NM. All authors proofread the manuscript.

## Conflicts of interest

The contact author has declared that none of the authors have any competing interests.

## Supplementary Material

EA-006-D5EA00117J-s001

## Data Availability

The optimized structures and calculation output files of all relevant compounds that support the findings of this manuscript will be available in the Zenodo repository: https://doi.org/10.5281/zenodo.17732259. Supplementary information (SI) is available. See DOI: https://doi.org/10.1039/d5ea00117j.
