# Supplementary material for: Acetyl peroxy radical-initiated oxidation of oxygenated monoterpenes: functional group effects on reaction pathways
Source: Environ Sci Atmos. 2025 Dec 1;6(2):224–37. doi: 10.1039/d5ea00117j (PMC12713733; doi:10.1039/d5ea00117j)
Supplement: EA-006-D5EA00117J-s001 [file EA-006-D5EA00117J-s001.pdf]

Supplementary Information (SI) for Environmental Science: Atmospheres.
This journal is © The Royal Society of Chemistry 2025

Supporting Information for Acetyl peroxy radical-initiated oxidation of oxygenated monoterpenes: functional group effects on reaction pathways

Ida Karppinen, Dominika Pasik, and Nanna Myllys

Lennard-Jones parameters for R-O reactions

Table S1: Lennard-Jones parameters σ and ϵ used in RRKM-ME calculations for ring-opening reaction reactant (MT-APR) and product (ring-opened MT-APR).

Monoterpenoid	MT-APR		Ring-opened MT-APR	
	σ (Å)	ϵ/k_B	σ (Å)	ϵ/k_B
Sabinol	7.76	649.28	7.93	661.7
Verbenol	7.88	647.13	8.08	655.56

Other calculated APR addition rates

In the manuscript, only the fastest APR addition rates are given. However, we have calculated rates for all the possible addition sites, also taking into consideration different sides of attack. Additional calculated rates that are not given in the manuscript are presented in Table S2. Reaction numbers in Table S2 corresponds to labeling in Fig. S1.

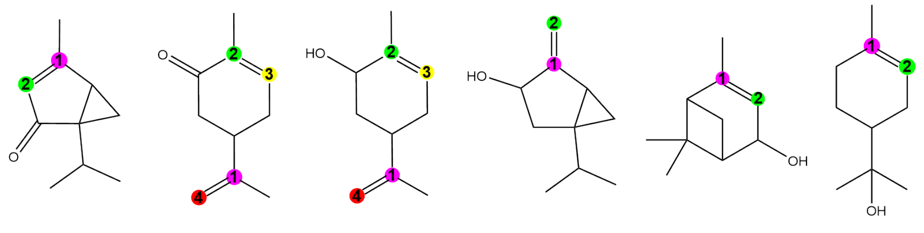

Figure S1: Studied monoterpenoid + APR reactions. APR addition sites are marked with numbers corresponding to Table S1.

Table S2: Calculated energy barrier heights (ΔE_1 in kcal mol^{-1}) and bimolecular MC-TST reaction rate coefficients (k_{bi} in cm^{-3} s^{-1}) for the rest of the APR + monoterpenoid accretion reactions at 298 K. Reaction numbers correspond to numbering in Fig. S1.

Monoterpenoid	Reaction	ΔE^{TS}	k_{bi}
α-Terpineol	R1	1.8	1.2×10^{-17}
Umbellulone	R1 (a)	7.1	4.9×10^{-21}
	R2 (a)	3.9	4.5×10^{-19}
	R2 (b)	4.6	1.7×10^{-19}
Carveol	R1	4.5	1.4×10^{-19}
	R2 (c)	0.51	6.6×10^{-17}
	R2 (d)	1.8	1.1×10^{-17}
	R3 (d)	1.7	3.4×10^{-16}
	R4	3.2	4.8×10^{-18}
Carvone	R1	4.4	2.9×10^{-19}
	R2	3.5	5.2×10^{-19}
	R4	2.4	3.5×10^{-17}
Sabinol	R1 (a,c)	4.4	3.4×10^{-19}
	R1 (a,d)	3.2	8.7×10^{-19}
	R1 (b,c)	2.0	7.8×10^{-18}
	R1 (b,d)	3.9	1.6×10^{-18}
	R2 (a,c)	0.4	3.6×10^{-16}
	R2 (a,d)	1.9	2.5×10^{-17}
	R2 (b,d)	2.0	2.5×10^{-17}
Verbenol	R1 (a,c)	1.6	8.5×10^{-18}
	R1 (a,d)	3.5	3.8×10^{-18}

Conformer sampling using GOAT

We conducted additional conformer sampling using GOAT to further investigate the reactions of APR with umbellulone and verbenol. For umbellulone, reaction R1 where APR addition occurs on the secondary ring side (reaction a) had a very high barrier (7.1 kcal/mol) compared to addition happening on the methyl group side (reaction b, 1.7 kcal/mol). There was also a discrepancy between reactions R1 and R2 as addition on the secondary ring side (reaction a) had a lower barrier than addition on the methyl group side (reaction b) for reaction R2 but for reaction R1 the opposite was observed. Therefore, we wanted to confirm that the lowest energy TS found with CREST for reaction R1 (a) would be correct and followed the same methodology as described in the manuscript for finding the TS conformers using GOAT. The amount of unique conformers found with CREST and GOAT of the reactant (umbellulone) and TS of reaction R1 (a) are shown in Table S3. As was stated in the manuscript, the lowest energy TS found using GOAT was the same structure that was obtained from CREST. GOAT was able to find one more conformer than CREST, however this conformer was a higher energy conformer and did not contribute to the rate meaningfully (see Table S4). The high barrier of reaction R1 (a) could be attributed to an unfavorable orientation of orbitals caused by the adjacent three-membered ring structure.

We further conducted conformer sampling using GOAT for all the studied reactions between APR and verbenol. For both reaction R1 and R2, APR attacks occuring from either the same side of the OH-group (reaction c) or from the opposite side (reaction d) had rates of the same order of magnitude despite there being a significant difference in reaction barriers. For example, a barrier of 1.6 kcal/mol and a reaction rate coefficient of 8.5×10^{-18} cm^{-3} s^{-1} was obtained for reaction R1 (a,c), whereas a barrier of 3.5 kcal/mol and a rate of 3.8×10^{-18} cm^{-3} s^{-1} was obtained for reaction R1 (a,d). The rates differ only by a factor of 2 yet the barriers differ by as much as 1.9 kcal/mol. In the manuscript, this discrepancy was associated with the different amount of unique conformers between different TS isomers. For APR attacks ocuring on the opposite side of the OH-group, more conformers within the 2.5 kcal/mol cutoff were found for the TS than attack happening on the OH-group side (see Table S3). Accordingly, our aim was to investigate whether GOAT could identify a greater number of conformers for the reactions in which CREST yielded only a limited set of conformers.

The amount of unique conformers after energy cutoff and filtering found with CREST and GOAT are presented in Table S3 for two different isomers of verbenol and all the TSs of reactions between verbenol and APR. For reactions c (APR attack occurs on the OH-group side), CREST and GOAT found the same amount of unique TS conformers. For reactions d (APR attack occurs on the opposite side of the OH-group), GOAT found a different amount of unique TS conformers than CREST, however the lowest energy TS conformers were the same. The rates for reactions R1 and R2 (a,c) were the same using conformers obtained from GOAT versus CREST and rates for reactions R1 and

R2 (a,d) were higher when calculated using conformers obtained with GOAT but still on the same order of magnitude. Conformer sampling conducted using GOAT validated our results for reactions between APR and verbenol, where the similar rates in spite of differences in the barriers can be attributed to the different amount of unique TS conformers. The difference in the number of unique conformers within the 2.5 kcal/mol cutoff likely arises from hydrogen bonding in the c reactions. These hydrogen-bonded transition state structures are strongly stabilized, lowering their relative energy. As a result, conformers without this stabilization often lie above the 2.5 kcal/mol threshold and are excluded after CREST/GOAT filtering. In the transition state of reaction d, hydrogen bonding is not as feasible since the OH group is positioned on the opposite side relative to the APR group. Therefore, even though the initial number of conformers is comparable between reactions c and d, fewer conformers remain within the cutoff for reactions c because of hydrogen bonding in the lowest energy conformers.

Table S3: Amount of unique conformers obtained using two different configurational search tools CREST and GOAT for all the studied APR addition reactions of verbenol and reaction R1 (a) of umbellulone.

Monoterpenoid	Structure	Unique conf. (CREST)	Unique conf. (GOAT)
Umbellulone	Reactant	3	3
	TS (R1 (a))	15	16
Verbenol	Reactant (R)	3	3
	Reactant (S)	2	2
	TS (R1 (a,c))	2	2
	TS (R1 (a,d))	15	19
	TS (R2 (a,c))	3	3
	TS (R2 (a,d))	18	17

Table S4: MC-TST reaction rate coefficients (k_{bi} in $\mathrm{cm}^{-3}\ \mathrm{s}^{-1}$) calculated using conformers obtained from CREST and GOAT for reactions of APR with umbellulone and verbenol at 298 K. Reaction numbers correspond to labeling in Fig. S1.

Monoterpenoid	Reaction	$k_{\mathrm{bi,CREST}}$	$k_{\mathrm{bi,GOAT}}$
Umbellulone	R1 (a)	4.9×10^{-21}	4.9×10^{-21}
Verbenol	R1 (a,c)	8.5×10^{-18}	8.5×10^{-18}
	R1 (a,d)	3.8×10^{-18}	5.4×10^{-18}
	R2 (a,c)	1.1×10^{-16}	1.1×10^{-16}
	R2 (a,d)	1.2×10^{-16}	1.7×10^{-16}

APR-addition with negative barrier

In Fig. S2 and S3 the energy diagrams of APR-addition reactions with sabinol (R2 (b,c)) and carveol (R3 (c)) are shown. Our calculations predicted negative barriers for these reactions. However the reactions proceed through the formation of a pre-reactive complex that is lower in energy than the reaction transition states. Reaction rate coefficients were calculated based on the negative barriers that were computed as the energy difference between the transition state and the free reactants.

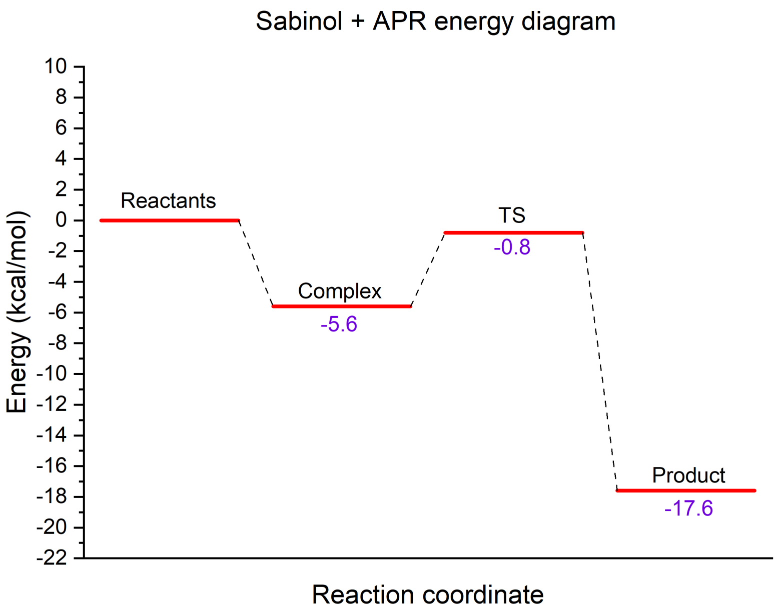

Figure S2: Energy diagram for sabinol + APR reaction R2 (b,c). Energies (in kcal/mol) are given relative to the energy of the free reactants.

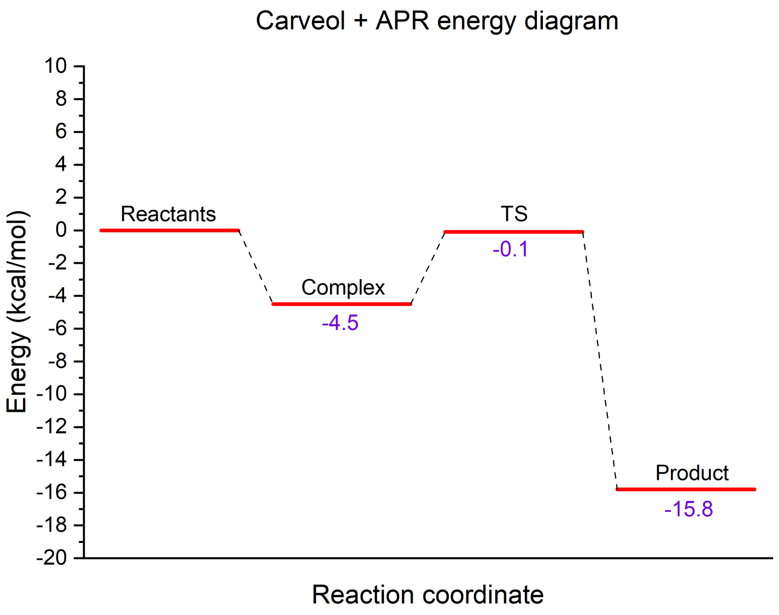

Figure S3: Energy diagram for carveol + APR reaction R3 (c). Energies (in kcal/mol) are given relative to the energy of the free reactants.

Stereochemistry of alkoxy radical β-scission reactions

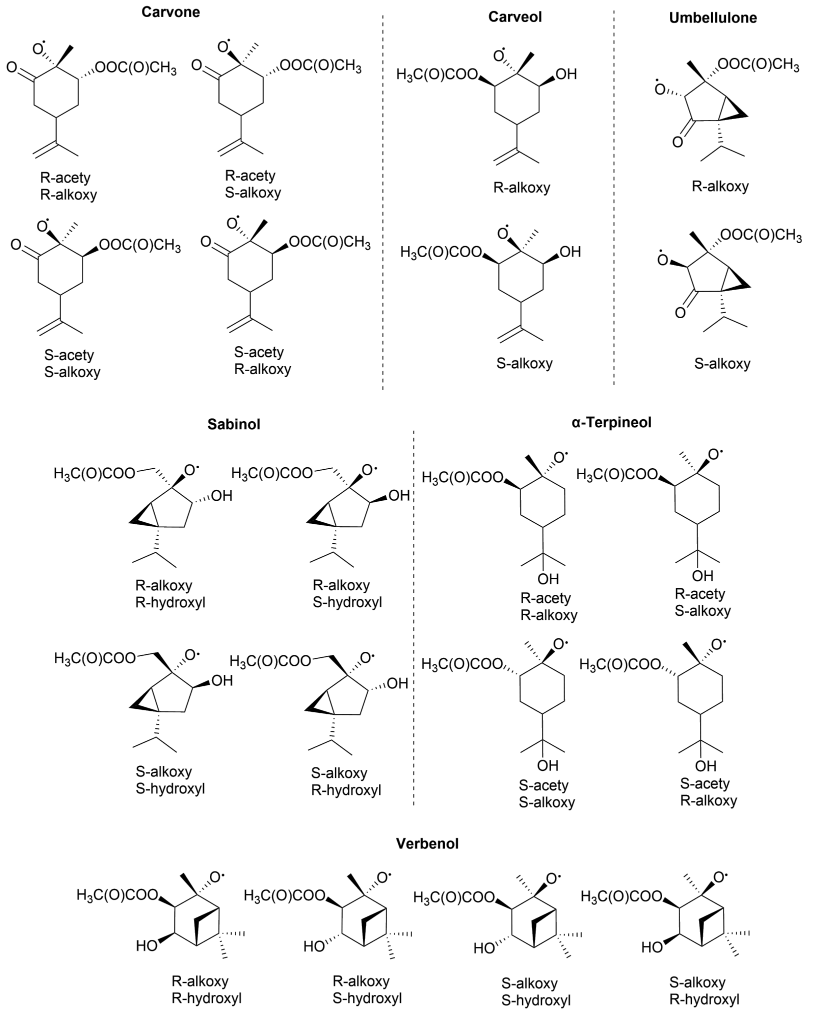

Figure S4: Molecular structures of investigated acetyl-alkoxyl stereoisomers of carvone, carveol, umbellulone, sabinol, α-terpineol and verbenol.
